# Methylation-Associated Differentiation Features Define Biological and Prognostic Heterogeneity in CMS4 Colorectal Cancer

**DOI:** 10.3390/ijms27177659

**Published:** 2026-08-26

**Authors:** Kaiyuan Xing, Liangshuang Li, Shuang Feng, Ting Yang, Yongjun He, Yingnan Ma, Wei Luo, Jiang Zhu

**Affiliations:** 1 School of Medical Informatics, Harbin Medical University, Daqing Campus, Daqing 163319, China; xky1922@163.com (K.X.); liliangshuang1997@163.com (L.L.); z15245098446@163.com (S.F.); 18161116991@163.com (T.Y.); hyj678117@163.com (Y.H.); mayingnan2020@outlook.com (Y.M.); 2 Key Laboratory of Frigid Zone Exercise Health Research and Translation in Heilongjiang Province, Xinyang Street 39, Daqing 163319, China

**Keywords:** CMS4, methylation, differentiation, scRNA-seq, colorectal cancer

## Abstract

Consensus molecular subtype 4 (CMS4) colorectal cancer (CRC) is associated with an aggressive clinical course and poor survival, yet the biological basis of heterogeneity within this subtype remains incompletely understood. DNA methylation is an epigenetic mechanism involved in transcriptional regulation, cellular differentiation, and colorectal tumorigenesis. Here, we integrated single-cell RNA sequencing (scRNA-seq), bulk data, and promoter DNA methylation data to characterize CMS4-associated cancer cell states and methylation-related features. Using the scAB algorithm, we integrated scRNA-seq with bulk CMS4 data and identified CMS4-related cells distributed across multiple patients. Single-cell analyses of cell–cell communication and transcriptional regulation revealed a CMS4-related cancer cell population characterized by macrophage migration inhibitory factor (MIF)-centered intercellular communication, enhanced caudal type homeobox 1 (CDX1) and Kruppel-like factor 5 (KLF5) regulon activity, and gene modules enriched in differentiation-related pathways. CytoTRACE analysis further stratified CMS4 cancer cells into poorly and well-differentiated states, yielding 802 differentially expressed genes (DEGs). Linking these differentiation-associated DEGs with bulk expression and promoter methylation data identified 218 methylation-associated DEGs showing significant inverse methylation expression correlations, suggesting a link between differentiation-related heterogeneity and promoter methylation. Univariable Cox regression followed by LASSO regression further prioritized eight genes for construction of the methylation and differentiation-related prognostic model (MeDiff-PM). MeDiff-PM consistently stratified overall survival in the TCGA CMS4 cohort and two independent validation cohorts, with cutoff-independent continuous Cox analyses further supporting its prognostic association across cohorts. And MeDiff-PM remained prognostically significant after adjustment for available clinical variables. High MeDiff-PM risk scores were associated with activation of P53, WNT, and ubiquitin-mediated proteolysis pathways and with consistent predicted drug response differences for compounds across three CMS4 cohorts. While individual in silico knockout analysis suggested links between MeDiff-PM genes and metallothionein-related and immune-associated transcriptional responses. Collectively, these findings indicate that methylation-associated differentiation features represent a molecular dimension of intra-CMS4 heterogeneity and provide a biologically informed framework for prognostic stratification within CMS4 CRC.

## 1. Introduction

Colorectal cancer (CRC) is the second leading cause of cancer-related deaths worldwide, posing a significant threat to global health [1]. Meanwhile, CRC is a heterogeneous disease, encompassing various subtypes. The consensus molecular subtype (CMS) classifier categorizes CRC into four subtypes: CMS1 (MSI Immune), CMS2 (Canonical), CMS3 (Metabolic), and CMS4 (Mesenchymal) [2]. Of them, CMS4 is characterized by prominent stromal activation and epithelial–mesenchymal transition (EMT). Compared to other subtypes, CMS4 is associated with a high recurrence rate, poor survival outcomes, and a limited response to traditional chemotherapy, making it a challenging point in current precision treatment [2,3]. Nevertheless, CMS4 patients do not experience uniform outcomes, indicating that clinically meaningful heterogeneity persists within this aggressive subtype. Defining the cellular and molecular features underlying this intra-CMS4 variation is therefore important for understanding disease progression and improving patient stratification.

Cancer cell differentiation plays a critical role in cancer diagnosis and treatment decisions. Poorly differentiated tumors are often associated with increased invasiveness and worse prognosis [4,5]. Specifically, the imbalance in cell differentiation status in CRC is closely linked to tumor progression, metastasis, and resistance, positioning it as a potential biomarker and therapeutic target. Analyzing the differentiation trajectories of cells across different cancer subtypes can reveal critical factors driving cancer progression [6,7]. For instance, Liang et al. reported that SRY-box transcription factor 9 (SOX9) enhanced CRC progression by inducing a stem cell-like program that inhibited intestinal differentiation [8]. Jiang et al. discovered that CRC subtypes exhibited distinct tissue differentiation patterns, with differentiation status closely linked to anti-tumor immune response and patient prognosis [9].

DNA methylation provides a plausible epigenetic link between cellular differentiation and persistent transcriptional states. Aberrant promoter methylation is common in CRC and can accompany altered expression of genes involved in tumor suppression, lineage identity, and cancer progression [10,11]. Abnormal methylation patterns have the potential to serve as valuable biomarkers for early detection and prognosis assessment in CRC [12,13]. Multi-omic studies further indicate that methylation alterations, copy-number changes, and transcriptional variation interact during CRC development and metastasis [14], while gene-specific promoter methylation can carry independent prognostic information [15].

Recent advances in single-cell RNA sequencing (scRNA-seq) have enabled the systematic portrayal of tumor heterogeneity, microenvironmental characteristics, and underlying molecular mechanisms at a single-cell resolution [16,17]. Meanwhile, the integration of scRNA-seq and bulk data has also made significant progress in the prognosis study of CRC. For example, by integrating scRNA-seq and bulk RNA-seq data, Xie et al. identified macrophage-related genes in CRC and developed a prognostic signature with strong predictive performance, which offered a robust tool for prognosis predictions to individual CRC patients [18]. Liu et al. assessed the immune profile of CRC patients and identified key prognostic biomarkers using both scRNA-seq and bulk RNA-seq [19]. Tao et al. explored mechanisms of circadian rhythm-related genes (CRDGs) and constructed a predictive model based on the combination of CRC scRNA-seq and bulk data, highlighting the potential of CRDGs as valuable biomarkers for prognostic prediction [20].

In this study, we first delineated the CMS4-associated cellular compartment and its intercellular and transcriptional regulatory features. We then identified genes that distinguished poorly differentiated from well-differentiated cancer cells and showed inverse relationships between promoter methylation and expression. These features converged on an eight-gene methylation and differentiation-related prognostic model (MeDiff-PM) that separated CMS4 tumors with distinct pathway activity, predicted drug responses, and clinical outcomes. Our findings support that methylation-associated differentiation features contribute to the diversity of malignant states observed within CMS4.

## 2. Results

### 2.1. Identification and Cellular Distribution of CMS4-Associated Cells

**Figure 1:**
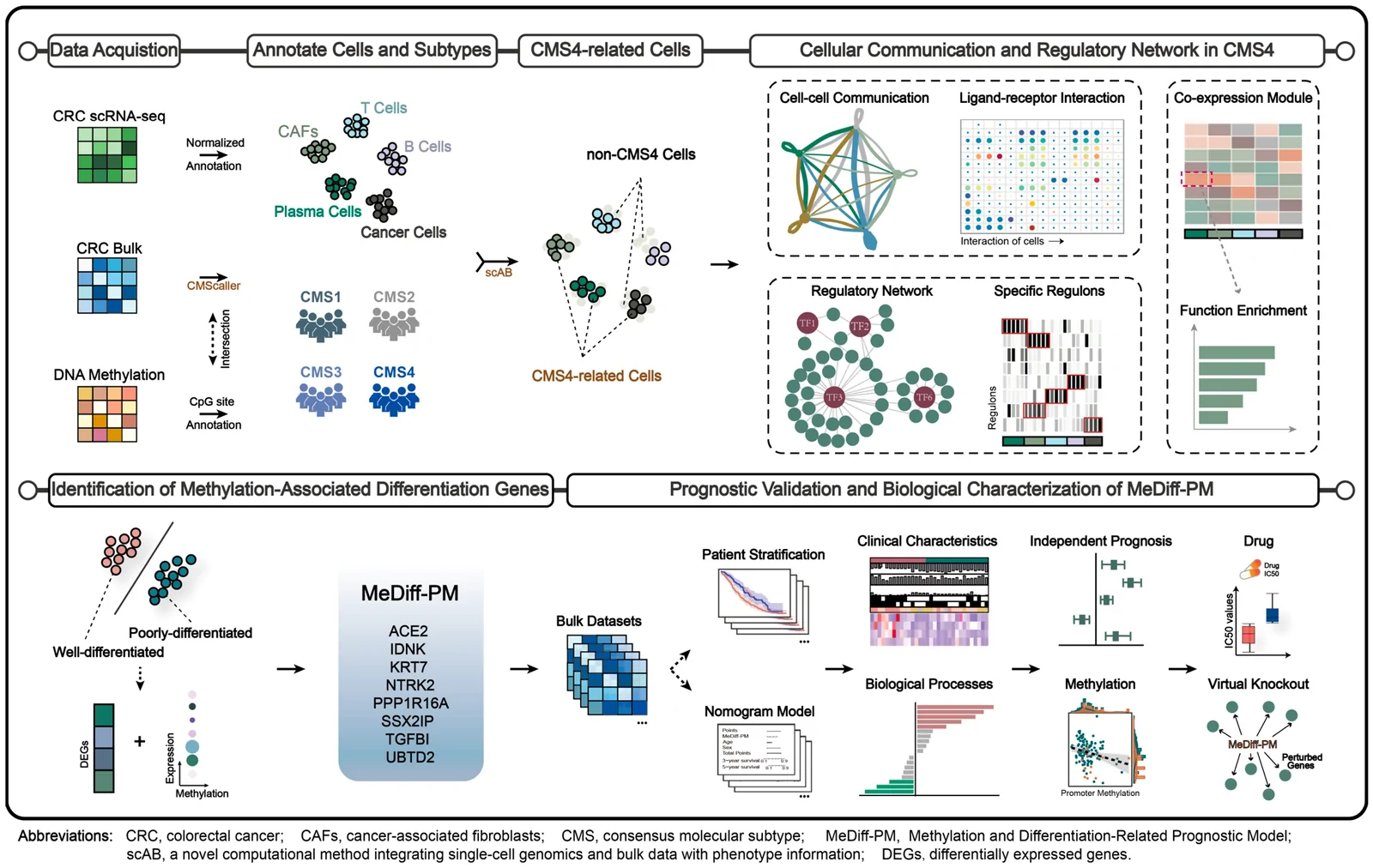
Overview of the study linking CMS4-associated cellular states, cancer cell differentiation, promoter methylation, and clinical outcome.

**Figure 2:**
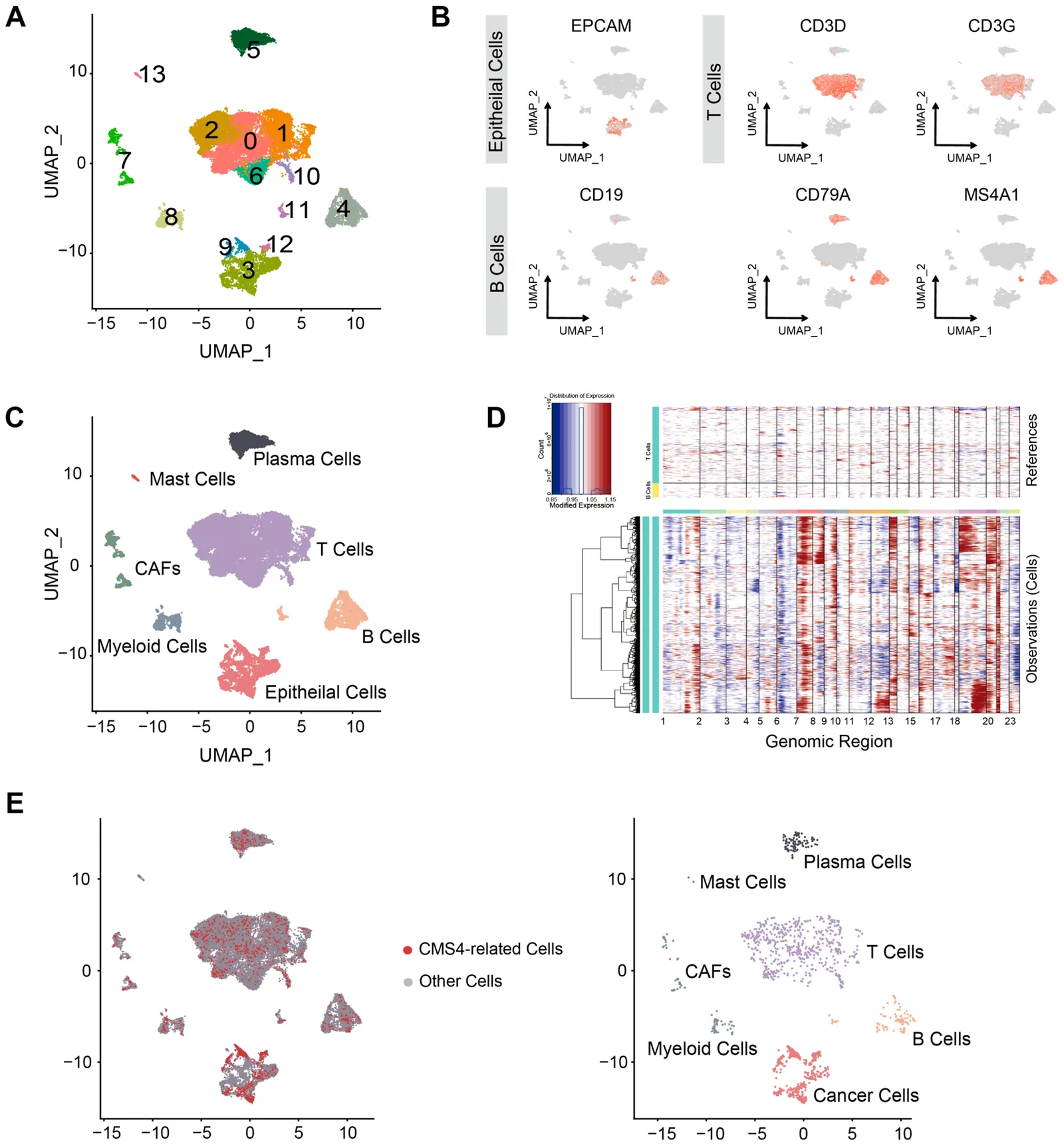
Cellular composition of consensus molecular subtype 4 (CMS4)-related cells in CRC. (**A**) UMAP plot of single cells clustered into 14 clusters, numbered from 0 to 13. (**B**) The expression of cell type (epithelial cells, T cells, and B cells) markers in cell clusters. Color intensity represents the relative expression level of each marker gene, with darker colors indicating higher expression and lighter colors indicating lower expression. (**C**) Cell type annotation with color labels for different types. (**D**) Selecting cancer cells in epithelial cells based on copy-number variations. (**E**) The distribution (**left**) and cell type composition (**right**) of CMS4 subgroups. CAFs, cancer-associated fibroblasts.

The overall study design is summarized in Figure 1. After quality control, the GSE200997 single-cell RNA sequencing (scRNA-seq) dataset comprised 27,453 high-quality cells with detectable expression of 16,181 protein-coding genes. Uniform manifold approximation and projection (UMAP) revealed 14 transcriptionally distinct cell clusters (Figure 2A) [21]. Based on canonical marker genes, these clusters were assigned to seven major cell types, including T cells, epithelial cells, B cells, plasma cells, cancer-associated fibroblasts (CAFs), myeloid cells, and mast cells (Figure 2B,C and Supplementary Figure S1A). Large-scale chromosomal copy-number variation (CNV) profiles further identified 4317 cancer cells within the epithelial compartment (Figure 2D).

In the TCGA dataset, 113 patients were classified as CMS4, which is characterized by distinct molecular and cellular features associated with poor prognosis in CRC. Integration of the subtype annotations with the scRNA-seq data identified a total of 1491 CMS4 subtype-related cells (Figure 2E and Supplementary Figure S1B). These cells were consistently distributed across multiple patients rather than dominated by a single individual, suggesting that the CMS4-associated transcriptional program is not driven by single-patient bias.

### 2.2. CMS4 Cancer Cells Exhibit MIF-Centered Communication and Distinct Regulatory Programs

**Figure 3:**
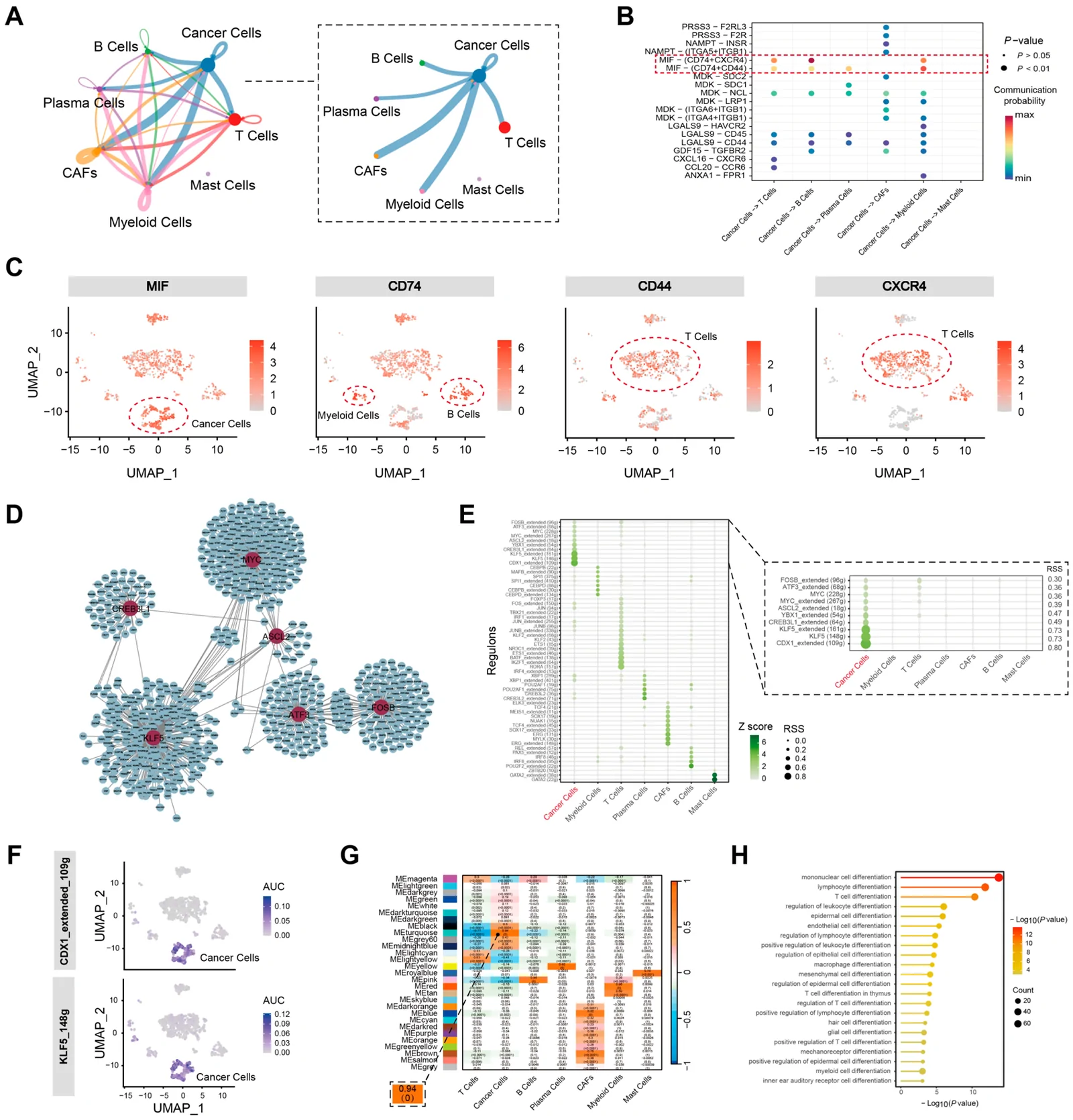
Intercellular communication and transcriptional regulatory features of CMS4 cancer cells. (**A**) Cell–cell interaction network of cancer cells and other cell types. The node size represents the number of cells, and the edge width represents the number of ligand–receptor pairs between cell groups. (**B**) Bubble plot of ligand–receptor pairs that mediate interactions of cancer cells and other cell types. Bubble size represents the statistical significance of the interaction, with larger bubbles indicating more significant interactions. Bubble color indicates the inferred communication probability, with redder colors corresponding to higher communication probabilities. (**C**) The expression levels of MIF, CD74, CXCR4, and CD44 in various cell types. (**D**) Gene regulatory networks composed of cancer cell-specific regulons. Brown (or cyan) means transcription factors (or target genes). (**E**) Cell type specificity of regulons ranked by the regulon specificity score (RSS). Dot size represents the RSS value, with larger dots indicating greater cell type specificity. (**F**) The activities of regulons in all cell types. The color scale represents AUCell scores (AUC), which quantify the enrichment of regulons within individual cells. Higher values indicate greater regulon activity. (**G**) The correlation between co-expression gene modules and cell types. (**H**) The results of the functional enrichment analysis of genes most relevant to cancer cells.

CMS4 cancer cells participated in an extensive intercellular communication network comprising 34 ligand–receptor pairs and 17 key signaling pathways (Figure 3A). Detailed information on these ligand–receptor interactions and signaling pathways is provided in Supplementary Table S1. The predicted ligand–receptor interaction patterns revealed a cell type-specific communication network involving CMS4 cancer cells and surrounding cell populations (Figure 3B). Macrophage migration inhibitory factor (MIF) emerged as a prominent cancer cell-derived signal within the CMS4 microenvironment, with the MIF-(CD74 + CXCR4) and MIF-(CD74 + CD44) ligand–receptor pairs showing relatively strong predicted interaction strengths. Consistent with this pattern, MIF was highly expressed in cancer cells, whereas the receptor components CD74 molecule (CD74), C-X-C chemokine receptor type 4 (CXCR4), and CD44 molecule (CD44) were predominantly detected in T cells and B cells (Figure 3C). Previous studies have linked elevated MIF expression to tumorigenesis and disease progression in CRC [22,23,24]. Together, these findings suggest that MIF-mediated signaling may contribute to communication between CMS4 cancer cells and other cell populations.

The cancer cells also exhibited a distinct transcriptional regulatory architecture. Based on identified co-expression modules between transcription factors (TFs) and their potential target genes, cancer cell state-specific gene regulatory networks (GRNs) were constructed (Figure 3D). Among 103 key cell type-associated regulons, 10 were preferentially enriched in cancer cells and collectively involved 701 predicted target genes (Figure 3E). The caudal type homeobox 1 (CDX1) and Kruppel-like factor 5 (KLF5) regulons showed the highest cancer cell specificity, with regulon-specific score (RSS) values of 0.80 and 0.73, respectively, and their activities were greatest in the cancer cells (Figure 3F). These findings identify CDX1- and KLF5-centered programs as prominent transcriptional regulatory features of CMS4 cancer cells and suggest that they may participate in maintaining their distinct molecular state.

A co-expression module containing 1020 genes showed the strongest association with cancer cells (r = 0.94, *p* < 0.001) (Figure 3G). The module was enriched in differentiation-related processes, including regulation of epithelial cell differentiation as well as mononuclear, lymphocyte, and T-cell differentiation (Figure 3H and Supplementary Table S2). This enrichment suggests that the cancer cell-associated module is linked to a broader differentiation-related transcriptional program.

### 2.3. Identification of Methylation-Associated Differentiation Genes with Prognostic Relevance in CMS4

**Figure 4:**
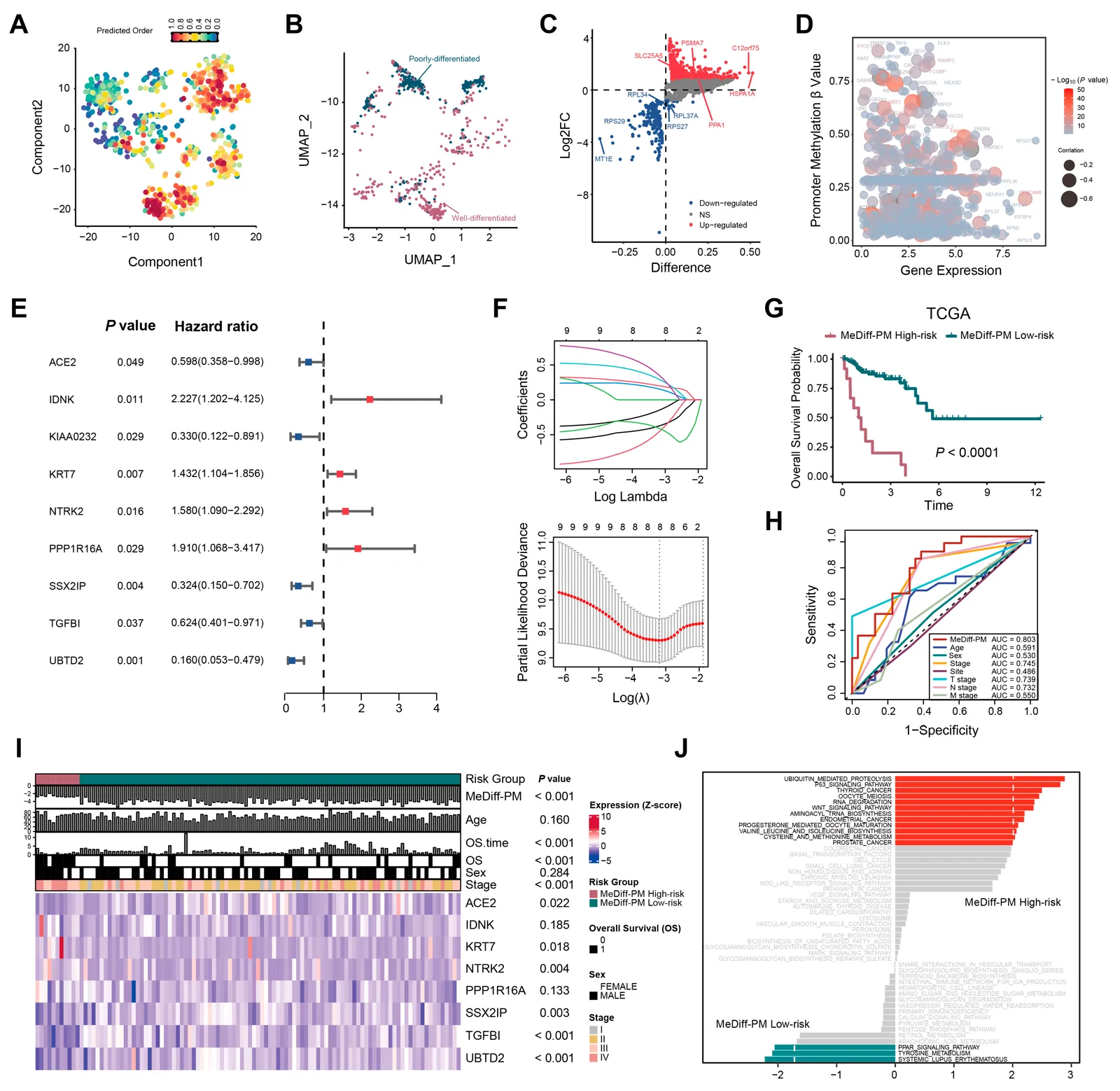
Identification of methylation-associated differentiation genes and prognostic characterization of MeDiff-PM in CMS4. (**A**) CytoTRACE predicted the differentiation potential of cancer cells in the scRNA-seq data. The higher the score, the lower the degree of cell differentiation. (**B**) Cancer cells were divided into poorly and well-differentiated groups based on the median CytoTRACE score. (**C**) Obtaining the differentially expressed genes (DEGs) between poorly and well-differentiated groups in the scRNA-seq data. The *x*-axis (“difference”) represents the difference in the proportion of cells expressing a given gene between poorly and well-differentiated groups (pct.1–pct.2). Higher values indicate greater cell population specificity. (**D**) Promoter methylation-expression correlations among differentiation-associated DEGs in TCGA CMS4 samples. Bubble size indicates the magnitude of the Spearman correlation coefficient, and bubble color represents the *p* value of the correlation analysis. (**E**,**F**) In the TCGA CMS4 cohort, overall survival (OS)-associated genes were identified using univariable Cox regression (**E**), followed by LASSO regression to select the MeDiff-PM genes (**F**). (**G**) Kaplan–Meier survival curves were used to compare the OS differences between high-risk and low-risk CMS4 patients stratified according to the optimal cutoff value of the MeDiff-PM risk score determined using the R package “survminer” in the TCGA CMS4 cohort. (**H**) ROC curves to evaluate the accuracy of MeDiff-PM and other clinical factors for predicting OS in TCGA CMS4 patients. (**I**) The differences in clinical characteristics and MeDiff-PM gene expression between MeDiff-PM subgroups. Values shown next to clinical variables and genes represent *p* values for comparisons between groups. Gene expression values were visualized as Z-scores. (**J**) The differences in gene sets activities between MeDiff-PM high- and low-risk groups in the TCGA dataset.

Cancer cells within the CMS4-associated population displayed variation in differentiation potential. Based on CytoTRACE (Cellular (Cyto) Trajectory Reconstruction Analysis using gene Counts and Expression) scores, 340 cancer cells were categorized into a poorly differentiated group and 339 into a well-differentiated group (Figure 4A,B). Comparison of these two cellular states identified 802 differentially expressed genes (DEGs) in the CMS4 scRNA-seq dataset, indicating substantial transcriptional differences associated with cancer cell differentiation (Figure 4C). Among these genes, 218 showed significant negative correlations between promoter methylation and gene expression levels in TCGA CMS4 samples, linking the differentiation phenotype to epigenetic variation (Figure 4D). Nine methylation-associated differentiation genes were associated with overall survival (OS) in the TCGA CMS4 cohort (*p* < 0.05) (Figure 4E). The LASSO regression analysis further selected eight genes with non-zero coefficients at the λ value corresponding to the minimum cross-validation error (0.04), which were combined into a methylation and differentiation-related prognostic model (MeDiff-PM) (Figure 4F): MeDiff-PM risk score = −0.154 × Exp(ACE2) + 0.203 × Exp(IDNK) + 0.158 × Exp(KRT7) + 0.299 × Exp(NTRK2) + 0.362 × Exp(PPP1R16A) − 0.378 × Exp(SSX2IP) − 0.468 × Exp(TGFBI) − 0.404 × Exp(UBTD2). Immunohistochemical images from the Human Protein Atlas (HPA) showed detectable protein expression for most MeDiff-PM genes in CRC tissues [25] (Supplementary Figure S2).

### 2.4. MeDiff-PM Identifies Clinically and Biologically Distinct CMS4 Groups

In the TCGA cohort, 113 CMS4 patients were classified into high- and low-risk groups using the cutoff threshold of −2.95, which was determined by the R package “survminer”. Patients in the high-risk group exhibited significantly shorter overall survival than those in the low-risk group (*p* < 0.0001) (Figure 4G). Comparison with available clinical factors showed that MeDiff-PM achieved the highest area under the receiver operating characteristic (ROC) curve (AUC = 0.803) for survival prediction (Figure 4H). The high- and low-risk groups also differed in the expression patterns of MeDiff-PM genes and in several clinical characteristics (Figure 4I).

Exploratory gene set variation analysis (GSVA) based on canonical pathway gene set collections from the MSigDB database suggested that MeDiff-PM risk stratification was associated with distinct pathway activity patterns [26]. Several pathways, including P53 signaling, WNT signaling, and ubiquitin-mediated proteolysis, showed higher activity scores in the high-risk group. These pathways are involved in cellular states relevant to cancer biology, including stemness maintenance, epithelial differentiation, and cell fate determination. Aberrant activation of WNT signaling has been reported to maintain stem-like states and impair normal epithelial differentiation, whereas p53 signaling is known to regulate cell fate decisions through modulation of proliferation, apoptosis, and stress responses [27,28,29]. In addition, ubiquitin-mediated proteolysis controls protein stability and regulates diverse cellular processes involved in tumor progression and cellular state maintenance [30]. In contrast, the low-risk group showed greater activity of the PPAR signaling pathway and tyrosine metabolism (Figure 4J). These results indicate that MeDiff-PM stratification was associated not only with survival differences but also with distinct biological processes within CMS4 tumors.

### 2.5. The Prognostic Association of MeDiff-PM Is Reproduced Across Independent Cohorts

**Figure 5:**
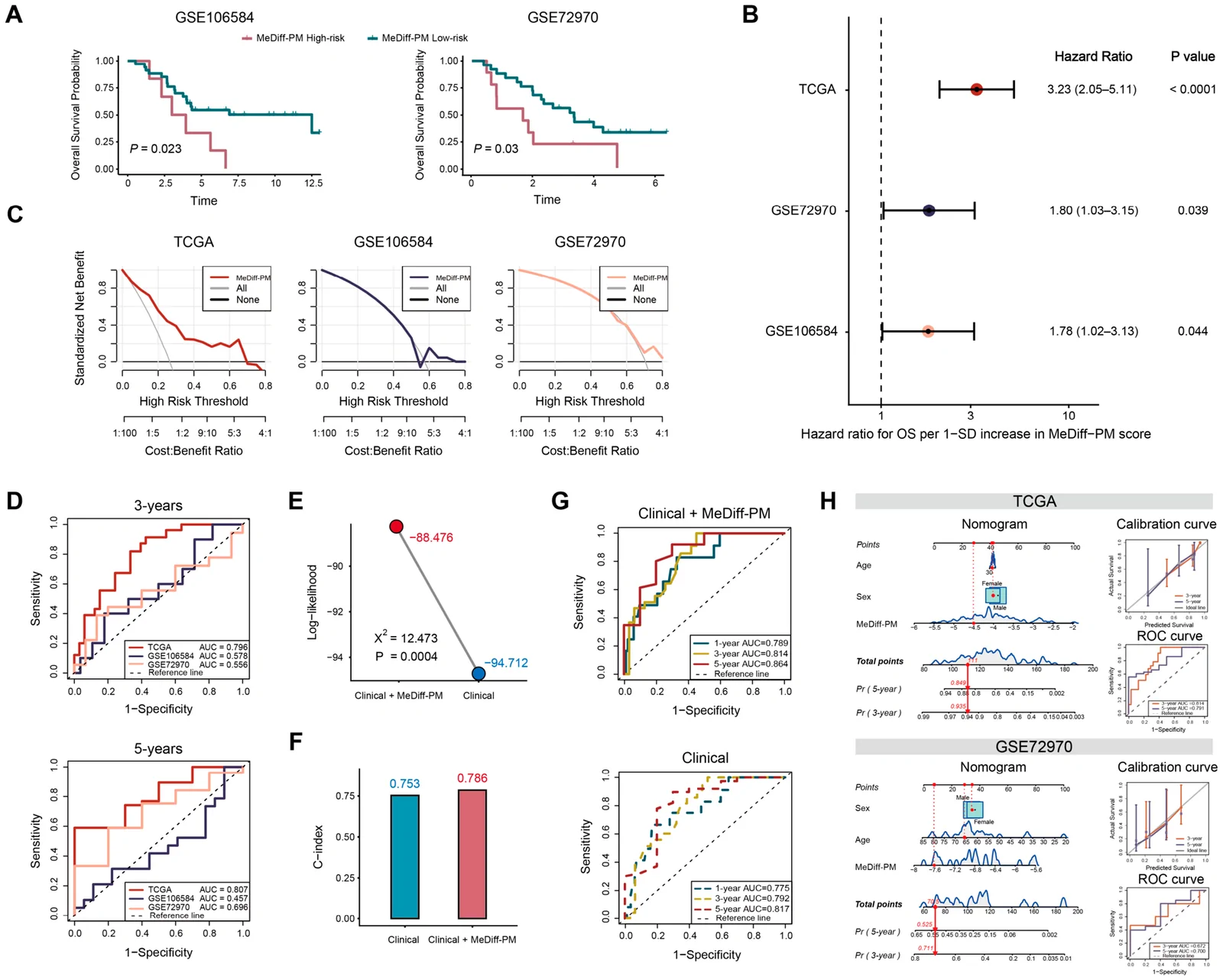
External prognostic evaluation of MeDiff-PM in validation datasets. (**A**) Comparing overall survival (OS) between the MeDiff-PM subgroups across two external bulk validation cohorts. Risk groups were defined using the optimal cutoff value of the MeDiff-PM risk score for within-cohort visualization of survival stratification. (**B**) Forest plot showing the association between continuous MeDiff-PM risk scores and OS. MeDiff-PM scores were standardized within each cohort, and hazard ratios (HRs) with 95% confidence intervals (CIs) were estimated using Cox proportional hazards regression per one-standard-deviation increase in the risk score. (**C**) Decision curves indicated that MeDiff-PM had good clinical utility. “All” and “None” represent the treat-all and treat-none strategies, respectively. (**D**) The receiver operating characteristic (ROC) curves evaluating the performance of MeDiff-PM in predicting 3- and 5-year OS of CMS4 patients. Evaluation of the incremental prognostic value of MeDiff-PM beyond clinical factors in the TCGA cohort. The clinical model included age, sex, and AJCC stage, and the combined model additionally incorporated MeDiff-PM. Model improvement was assessed by (**E**) likelihood ratio testing, (**F**) Harrell’s C-index comparison, and (**G**) time-dependent ROC analysis. (**H**) Nomograms integrating MeDiff-PM risk scores with available clinical variables, together with calibration curves and ROC analyses used to assess their predictive performance.

The prognostic association of MeDiff-PM was further evaluated in two independent external bulk cohorts, GSE106584 and GSE72970. For within-cohort visualization of survival stratification, patients were divided into high- and low-risk groups based on the optimal cutoffs. Kaplan–Meier analysis showed that patients in the high-risk group had significantly shorter overall survival than those in the low-risk group in both validation cohorts (*p* < 0.05) (Figure 5A). To further assess the prognostic association of MeDiff-PM independently of survival-derived cutoffs, MeDiff-PM was additionally evaluated as a continuous variable using Cox proportional hazards regression after within-cohort standardization of the risk scores in the TCGA cohort and two validation cohorts. Higher MeDiff-PM risk scores were consistently associated with increased mortality risk across all three cohorts (Figure 5B). Decision curve analysis (DCA) suggested that MeDiff-PM provided a positive net benefit (Figure 5C). ROC analyses further indicated generally favorable performance for predicting 3- and 5-year OS for CMS4 patients (Figure 5D). Although predictive performance varied across cohorts, the concordant Kaplan–Meier and cutoff-independent continuous Cox analyses supported the reproducibility of the prognostic association of MeDiff-PM across cohorts.

### 2.6. MeDiff-PM Provides Prognostic Information Beyond Available Clinical Variables

The association between MeDiff-PM risk scores and overall survival remained significant after adjustment for available clinical characteristics in the TCGA and GSE72970 cohorts (Table 1). The GSE106584 dataset was excluded from this analysis due to the lack of age and sex information. In the TCGA cohort, the multivariable model included age, sex, AJCC stage, and MeDiff-PM. In GSE72970, T and N stages were used as surrogate indicators because complete AJCC stage information was unavailable. Across all cohorts, MeDiff-PM consistently acted as an independent prognostic factor (*p* < 0.05), with patients in the high-risk group showing increased mortality risk (hazard ratio (HR) > 1.00). When treated as a continuous variable, the MeDiff-PM risk score remained significantly associated with OS in the TCGA cohort after adjustment for age, sex, and AJCC stage (univariable Cox: HR = 5.463, 95% CI = 2.820–10.584, *p* < 0.001; multivariable Cox: HR = 4.192, 95% CI = 2.142–8.205, *p* < 0.001), indicating that the prognostic information provided by MeDiff-PM was not solely dependent on dichotomization by the optimal cutoff.

To further evaluate whether MeDiff-PM provided additional prognostic value beyond clinical characteristics, we compared a clinical model based on age, sex, and AJCC stage with a combined model incorporating MeDiff-PM in the TCGA cohort. The addition of MeDiff-PM significantly improved model performance, as demonstrated by likelihood ratio testing ($\chi^{2}$ = 12.473, *p* = 0.0004) (Figure 5E). Furthermore, the C-index increased from 0.753 for the clinical model to 0.786 for the combined model (Figure 5F). Time-dependent ROC analysis further demonstrated improved prognostic discrimination after incorporating MeDiff-PM at 1-, 3-, and 5-year survival (combined model vs. clinical model AUC: 0.789 vs. 0.775, 0.814 vs. 0.792, and 0.864 vs. 0.817, respectively) (Figure 5G).

Comprehensive nomograms incorporating MeDiff-PM risk scores and the common clinical variables (age and sex) were constructed to predict 3- and 5-year overall survival probabilities of CMS4 patients. Their predictive performance was evaluated using calibration curves and ROC analyses. The calibration curves showed general agreement between the predicted and observed survival rates. The ROC curves and AUC values further indicated favorable discrimination at the evaluated time points (Figure 5H). These findings suggest that integrating MeDiff-PM with clinical variables may improve prognostic risk estimation in CMS4 patients, although further validation in larger and prospective cohorts is required.

### 2.7. Exploratory Analysis of Predicted Drug Response Associated with MeDiff-PM Risk Stratification

**Figure 6:**
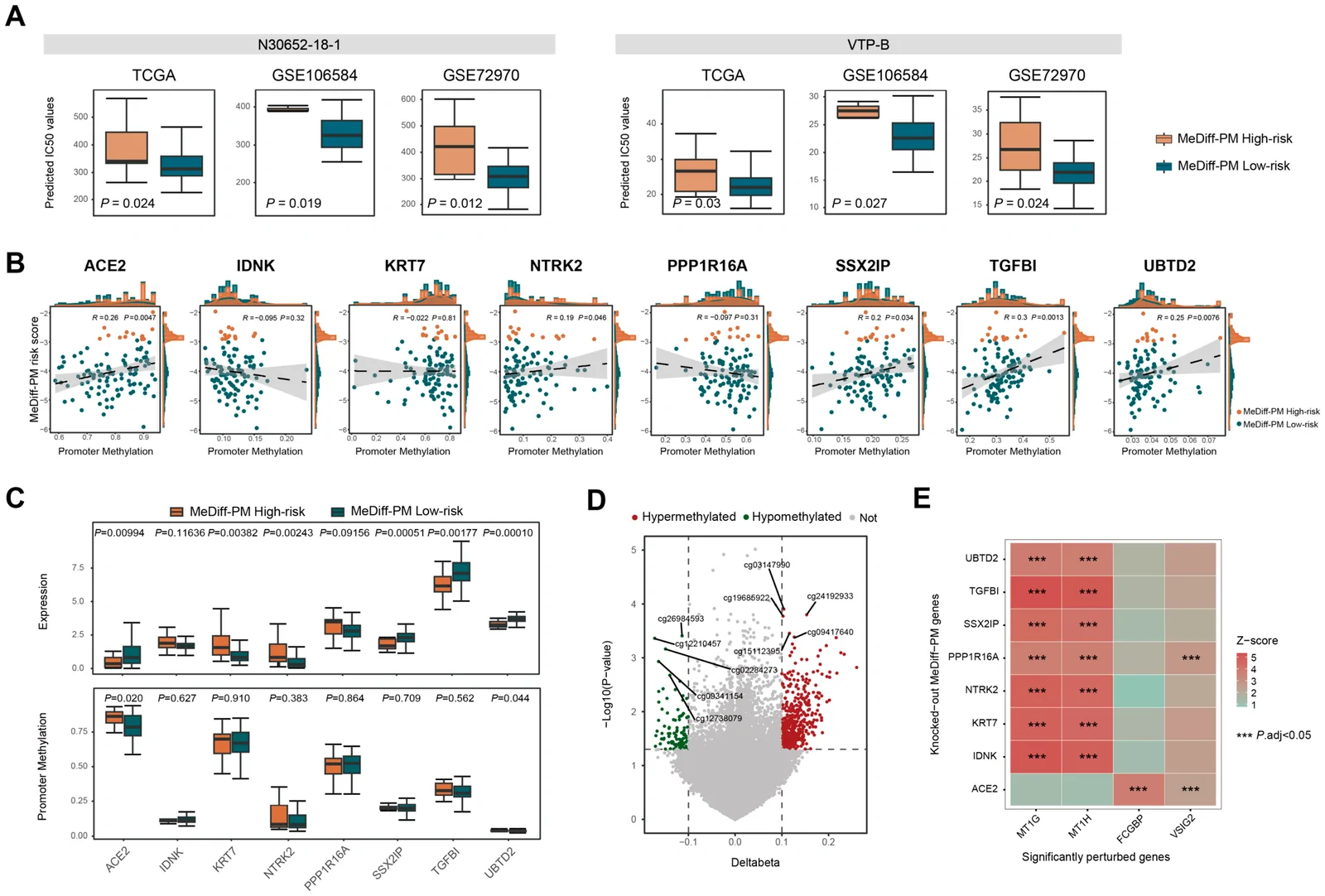
Characterization of promoter methylation patterns and predicted drug response associated with MeDiff-PM. (**A**) Compounds showing consistent directional differences in predicted IC50 values between the MeDiff-PM high- and low-risk groups across all three CMS4 bulk cohorts. (**B**) Correlations between promoter methylation levels of MeDiff-PM genes and MeDiff-PM risk scores in the TCGA dataset. The dashed line indicates the fitted linear regression line. (**C**) The differences in gene expression (**upper**) and promoter methylation levels (**lower**) of MeDiff-PM genes between the MeDiff-PM high-risk and low-risk groups in the TCGA cohort. (**D**) Differentially methylated probes between MeDiff-PM high-risk and low-risk groups. (**E**) Heatmap showing significantly perturbed genes after individual in silico knockout of each MeDiff-PM gene. Color intensity represents the perturbation Z-score.

Exploratory analysis of predicted drug response revealed that MeDiff-PM risk stratification was associated with distinct drug response patterns. Across the TCGA CMS4 cohort and two independent CMS4 validation cohorts (GSE106584 and GSE72970), two compounds, N30652-18-1 and VTP-B, showed consistent differences in predicted IC50 values between the MeDiff-PM high- and low-risk groups (Figure 6A). Both compounds exhibited higher predicted IC50 values in the high-risk group across all three cohorts, suggesting reduced sensitivity associated with the MeDiff-PM high-risk phenotype.

### 2.8. Promoter Methylation Features Associated with MeDiff-PM Risk Stratification

In the TCGA cohort, promoter methylation levels of most MeDiff-PM genes were positively correlated with the MeDiff-PM risk scores, indicating that higher risk scores were associated with coordinated differences in promoter methylation patterns of these genes (Figure 6B). Six of the eight MeDiff-PM genes showed significant expression differences between the MeDiff-PM high- and low-risk groups, whereas ACE2 and UBTD2 also exhibited significant differences in promoter methylation levels (Figure 6C). Genome-wide comparison between the MeDiff-PM high- and low-risk groups identified 552 differentially methylated probes (DMPs). These DMPs were significantly enriched in the promoter regions of MeDiff-PM genes (hypergeometric *p* = 0.026) and showed tissue-associated distribution patterns (Figure 6D). Together, these findings indicate that the transcriptional differences between MeDiff-PM risk groups are accompanied by distinct promoter methylation features.

### 2.9. MeDiff-PM Genes Link to Metallothionein-Related Transcriptional Responses

Virtual knockout analysis revealed a partially convergent transcriptional response associated with MeDiff-PM genes in CMS4 cancer cells from the scRNA-seq data. Among the eight MeDiff-PM genes, perturbation of seven genes consistently identified MT1G and MT1H as significantly perturbed genes, whereas perturbation of the ACE2 mainly affected FCGBP and VSIG2 (Figure 6E). MT1G and MT1H belong to the metallothionein family, members of which are broadly involved in metal ion homeostasis, oxidative stress responses, and cellular protection. FCGBP and VSIG2 are associated with epithelial, mucosal, and immune-related features. These findings suggest that MeDiff-PM genes may be linked to metallothionein-related and epithelial/immune-associated transcriptional responses in CMS4 cancer cells.

## 3. Discussion

Colorectal cancer (CRC) is a highly heterogeneous malignancy comprising molecular subtypes with distinct biological features and clinical outcomes [31]. The consensus molecular subtype 4 (CMS4) is characterized by robust stromal response, extensive remodeling of the tumor microenvironment, and generally unfavorable clinical outcomes [2]. Although CMS4 CRC is generally associated with poor prognosis, clinical outcomes still vary among patients within this subtype, suggesting that additional biological features may contribute to intra-subtype heterogeneity. Cancer-cell differentiation is closely associated with tumor aggressiveness, metastatic potential, and patient prognosis [32]. As the core epigenetic mechanism, DNA methylation plays an essential role in the precise regulation of gene expression and may also be crucial for driving cell fate determination and differentiation [33]. In this context, single-cell RNA sequencing (scRNA-seq) has emerged as a powerful tool for unraveling tumor heterogeneity, as well as for investigating cellular differentiation and cancer prognosis.

The communication and regulatory features observed in CMS4 cancer cells provide additional biological context for this variation. MIF expression in malignant cells, together with CD74, CXCR4, and CD44 expression in immune populations, suggests a possible route through which CMS4 cancer cells may interact with the surrounding immune compartment. In parallel, the preferential activity of CDX1 and KLF5 regulons highlights cancer cell-enriched transcriptional regulatory programs within the CMS4 malignant population. The enrichment of genes in the cancer cell-associated co-expression module further suggests that this module is associated with differentiation in CMS4. In particular, cancer cells with different differentiation potentials displayed transcriptional differences, and a subset of these differentiation-associated genes showed negative promoter methylation expression relationships, supporting a potential epigenetic component of differentiation-related molecular variation in CMS4. Although these findings do not establish causal regulation, they indicate that intercellular signaling, transcription-factor activity, promoter methylation, and differentiation-associated transcriptional programs are interconnected features of the CMS4 ecosystem. Based on these methylation-associated differentiation genes, a methylation and differentiation-related prognostic model (MeDiff-PM) was developed. MeDiff-PM provided a patient-level representation of these methylation-associated differentiation features. DNA methylation represents an important epigenetic mechanism involved in transcriptional activity, particularly through promoter methylation-mediated alteration of gene expression [34,35]. Among the MeDiff-PM genes, TGFBI expression has been reported to be regulated by DNA methylation in CRC [12]. The aberrant methylation of the ACE2 promoter has been associated with high ACE2 expression in human disease contexts [36]. These findings support the rationale for integrating DNA methylation and transcriptional features in the construction of MeDiff-PM.

Several MeDiff-PM genes have been implicated in regulating tumor cell states, providing biological support for the association between MeDiff-PM and differentiation-related phenotypes. Among the eight model genes, KRT7, NTRK2, and TGFBI have been previously reported to be associated with CRC. KRT7 is an epithelial cytokeratin that reflects epithelial lineage identity and differentiation status. Its aberrant expression, regulated by KRT7-AS, plays a pivotal role in promoting CRC cell migration [37,38,39,40]. NTRK2/TRKB signaling, originally characterized as a regulator of differentiation and survival during development, and NTRK2 has been proposed as a potential biomarker in CRC [41,42]. TGFBI is a TGF-β-inducible extracellular matrix protein involved in cell adhesion and matrix remodeling. Importantly, CMS4 tumors are characterized by enhanced TGF-β signaling and stromal activation, suggesting that TGFBI may represent a potential molecular link between MeDiff-PM and CMS4-associated mesenchymal states [2,43]. And it is a promising candidate biomarker for the early diagnosis of CRC. Together, these findings suggest that MeDiff-PM may capture differentiation-associated tumor cell states rather than merely reflecting prognostic differences.

High-risk patients showed shorter survival and greater activity of P53, WNT, and ubiquitin-mediated proteolysis pathways, whereas low-risk patients showed enrichment of metabolic programs. The prognostic association of MeDiff-PM was reproduced across two independent external cohorts, and cutoff-independent analyses of the continuous risk score further showed consistently positive hazard ratios across the TCGA and validation cohorts. And the MeDiff-PM risk scores remained significantly associated with overall survival after adjustment for available clinical variables, supporting its independent prognostic relevance. Furthermore, incorporation of MeDiff-PM significantly improved prognostic discrimination beyond clinical factors in the TCGA cohort, suggesting that MeDiff-PM may provide complementary prognostic information among CMS4 patients, although its predictive performance varied across validation cohorts. Notably, a relatively low 5-year AUC was observed in the GSE106584 cohort. This finding may be attributable to the small number of death events occurring beyond the 5-year time point (*n* = 4), which may reduce the stability of time-dependent ROC estimation. Therefore, further validation in larger cohorts with longer follow-up is warranted to better evaluate the long-term predictive performance of MeDiff-PM. Exploratory predicted drug response and virtual knockout analyses further suggested that MeDiff-PM risk stratification may be associated with distinct predicted drug response patterns and biological features in CMS4.

Several limitations of this study should be acknowledged. First, although OncoPredict has been widely applied to infer drug sensitivity [44,45,46], its model is trained using GDSC cell lines that lack stromal and immune components. This limitation is particularly relevant for CMS4, which is characterized by prominent stromal activation, and may limit the reliability of drug sensitivity predictions. Future validation using patient-derived organoids, ex vivo drug response assays, or in vivo models is required. Second, MeDiff-PM was developed using bulk tumor datasets, and although the model genes were derived from cancer cell differentiation states identified by scRNA-seq, potential influences from tumor microenvironmental composition cannot be completely excluded. In addition, direct protein-level validation and functional experiments would provide additional support for the biological roles of MeDiff-PM genes. Third, MeDiff-PM was developed in a relatively limited CMS4 cohort, which may affect model stability and increase the potential risk of overfitting. Furthermore, the cutoff-based stratification resulted in an imbalanced distribution of patients between risk groups, which may influence clinical interpretation. Although MeDiff-PM showed consistent prognostic performance across multiple independent cohorts, larger CMS4 cohorts are needed to further validate its robustness, generalizability, and clinical applicability.

In conclusion, our findings suggest that methylation-associated differentiation features are linked to biological and prognostic heterogeneity within CMS4 CRC. Rather than representing a uniform molecular subtype, CMS4 tumors appear to contain distinct cancer cell-associated transcriptional and epigenetic features related to differentiation status and patient outcome. MeDiff-PM summarized this methylation and differentiation-related variation at the bulk-tumor level and was associated with survival differences across multiple cohorts. Differences in pathway activity, promoter methylation patterns, and predicted drug-response further indicate that MeDiff-PM-defined groups may capture biologically distinct features within CMS4 tumors. These findings provide a biological framework for understanding intra-CMS4 heterogeneity and warrant further experimental and clinical validation.

## 4. Materials and Methods

### 4.1. Data Sources and Preprocessing

The colorectal cancer (CRC) single-cell RNA sequencing (scRNA-seq) data of GSE200997 were downloaded from the Gene Expression Omnibus (GEO) database (https://www.ncbi.nlm.nih.gov/geo/ (accessed on 13 July 2023)), which contains 31,586 cells from 16 patients (Table 2) [47]. The dataset was selected because it provides complete raw unique molecular identifier (UMI) count matrices and comprehensive annotations, making it suitable for characterizing the cellular landscape of the CMS4 and subsequent downstream analyses. Quality control procedures were implemented for both cells and genes in the raw expression data using the R package “Seurat” as follows: (1) Cells with fewer than 200 detected genes or those with more than 15% of mitochondrial genes relative to total gene expression were excluded to eliminate low-quality or stressed cells. (2) Genes expressed in fewer than 3 cells were removed to minimize the impact of lower-expressed genes. (3) Mitochondrial and ribosomal genes were filtered out to focus exclusively on protein-coding genes [47,48]. Gene expression data were normalized using the LogNormalize method. Highly variable genes were identified using the variance stabilizing transformation (vst) method. The data were scaled and principal component analysis (PCA) was performed using variable genes. The first 50 principal components were used for downstream analyses. A shared nearest-neighbor graph was constructed and distinct cell clusters were identified using the Louvain algorithm implemented in FindClusters. The corresponding cell type labels were assigned to each cluster based on known marker genes provided by Che et al. [49]. Finally, cancer cells were identified using the inferCNV (https://github.com/broadinstitute/inferCNV (accessed on 10 August 2023)), with gene filtering threshold set at 0.1 and HMM = FALSE, while all other parameters were kept at default settings. The large-scale chromosomal copy-number variation (CNV) profiles were inferred by comparing epithelial cells against reference cells (T cells and B cells). CNV scores were calculated at the chromosomal region level after smoothing across adjacent genes. Epithelial cells exhibiting broad and consistent chromosomal CNV deviations relative to reference cells were classified as cancer cells [50,51].

The DNA methylation (450K), bulk RNA-seq data, and corresponding clinical information of CRC samples were retrieved from the TCGA dataset. We selected samples that contained all the above-mentioned data. The DNA methylation values were described as β values, ranging from 0 to 1. Promoter regions were defined as CpG probes annotated to the TSS200 (transcription start site) and TSS1500 regions. The average methylation level at the CpG sites in the promoter region was considered the promoter methylation level of the corresponding gene. Detailed annotation information of promoter-associated CpG probes for MeDiff-PM genes is provided in Supplementary Table S3. In addition, the expression and clinical data from 2 bulk datasets were acquired as validation datasets, including GSE106584 [52], GSE72970 [53,54] (Table 2). Each dataset was analyzed independently using normalized expression matrices, with log2 transformation applied when necessary.

### 4.2. Identification of CMS4-Related Cells and Matched Methylation Profiles

To identify consensus molecular subtype 4 (CMS4) patients across the 3 bulk datasets (TCGA, GSE106584, and GSE72970), CMS classification was performed independently for each cohort using the CMScaller R package [55]. CMScaller assigns CMS labels (CMS1, CMS2, CMS3, and CMS4) based on the correlation between individual sample expression profiles and predefined CMS reference centroids, enabling single-sample CMS classification (Table 2). Samples classified as CMS4 were selected for subsequent analyses. Since CMS assignment was performed independently within each cohort, no cross-cohort batch correction was applied. The promoter methylation and gene expression data of CMS4 samples in the TCGA dataset were extracted based on the classification results. We then integrated scRNA-seq data, bulk data and CMS4 subtype information from the TCGA dataset using the scAB algorithm to obtain cell subgroups associated with CMS4 [56]. The scAB algorithm was performed according to the original publication using default parameters. Briefly, scAB constructs a cell-to-sample similarity matrix based on pairwise Pearson correlations between individual cells and bulk samples, followed by knowledge- and graph-guided matrix factorization to identify phenotype-associated cell patterns. Cells associated with CMS4 were identified based on the inferred cell loading matrix (H) using the adaptive thresholding strategy implemented in scAB.

### 4.3. Characterization of CMS4 Cellular Communication and Regulatory Programs

Based on the CMS4 scRNA-seq data, cell–cell communication analysis was conducted to explore the crosstalk between cancer cells and other cell types in the CMS4, and to identify ligand–receptor pairs that mediate the strong regulation of cancer cells. Next, we developed a specifically active gene regulatory network (GRN) for the cancer cells of CMS4 to determine key regulons (consisting of the transcription factors (TFs) and their putative target genes) using the SCENIC method [57]. To further elucidate the functional differences in key genes across various cell types, we applied weighted gene co-expression network analysis (WGCNA) to pinpoint essential gene modules associated with each cell type [58]. Enrichment analysis for gene modules was performed using the R package “clusterProfiler” [59].

### 4.4. Identification of Methylation-Associated Differentiation Features and Construction of MeDiff-PM

The differentiation potential of cancer cells in the CMS4 scRNA-seq data was evaluated using the CytoTRACE method [60]. Based on the CytoTRACE scores, cancer cells were classified into well-differentiated (with CytoTRACE scores < 0.5) and poorly differentiated (with CytoTRACE scores ≥ 0.5) groups. Differential expression analysis was performed between the well- and poorly differentiated groups using the FindAllMarkers function in the package “Seurat”. Differentially expressed genes (DEGs) were identified using the Wilcoxon rank-sum test (*p* < 0.05 and |log_2_FC| > 0.8). The differentiation-associated DEGs identified from cancer cells at the single-cell level were subsequently matched with their corresponding gene expression and promoter methylation profiles in TCGA CMS4 bulk tumors. Promoter methylation information was incorporated as a biological filtering criterion to refine methylation-associated DEGs prior to model construction. All subsequent methylation expression integration and model construction procedures were performed in the TCGA CMS4 cohort. Spearman correlation analysis was performed between promoter methylation levels and mRNA expression levels for each DEG. Genes showing a significant negative correlation (*p* < 0.05) were defined as key methylation-associated DEGs. Next, univariable Cox regression analysis was performed as an initial screening step to identify genes associated with OS (*p* < 0.05) from key methylation-associated DEGs. Least Absolute Shrinkage and Selection Operator (LASSO) Cox regression was performed using the R package “glmnet” to identify model genes. Ten-fold cross-validation was applied to determine the optimal penalty parameter (λ), and genes with non-zero coefficients at the λ value corresponding to the minimum cross-validation error were retained for construction of the MeDiff-PM:

(1) $$MeDiff-PM\;\mathrm{risk}\;\mathrm{score}\;=\sum_{i=1}^{n}{\mathrm{coef}}_{i}{\mathrm{Exp}}_{i}$$

where ${\mathrm{coef}}_{i}$ represents the Lasso regression coefficient of gene i, n is the number of model genes, and ${\mathrm{Exp}}_{i}$ is the expression value of gene i in the sample.

### 4.5. Assessment of the Prognostic Relevance of MeDiff-PM

MeDiff-PM risk scores were calculated for CMS4 patients in the TCGA and two independent GEO external validation cohorts. Cohort-specific optimal cutoffs were determined using the surv_cutpoint function in the R package “survminer”, which identifies the threshold that maximizes the separation of survival between groups [61]. These cutoff-based analyses were used for within-cohort visualization of survival stratification. Based on the best cutoff, CMS4 patients were divided into high-risk and low-risk groups. Then the differences in overall survival (OS) between high- and low-risk groups were assessed by Kaplan–Meier (K-M) analysis and the log-rank test. To further evaluate the prognostic relevance of MeDiff-PM independently of survival-derived cutoffs, MeDiff-PM risk scores were additionally analyzed as a continuous variable using Cox proportional hazards regression. Because the TCGA and GEO datasets were generated using different transcriptomic platforms and exhibited different numerical distributions of MeDiff-PM risk scores, the risk scores were standardized within each cohort. Hazard ratios (HRs) and 95% confidence intervals (CIs) were estimated per one-standard-deviation increase in the MeDiff-PM risk score.

The receiver operating characteristic (ROC) curves were utilized to validate the predictive performance of MeDiff-PM at 3- and 5-year follow-up. The decision curve analysis (DCA) examined the clinical application value of MeDiff-PM. Univariate and multivariate Cox regression analyses were performed to determine whether MeDiff-PM was independent of other clinical characteristics (e.g., sex, age and stage) for CMS4 patients. Two Cox proportional hazards models were constructed in the TCGA cohort to evaluate the incremental prognostic value of MeDiff-PM beyond clinical factors. The clinical model included age, sex, and AJCC stage, whereas the combined model additionally incorporated the MeDiff-PM risk scores. The performance of the two models was compared using likelihood ratio tests, concordance index (C-index), and time-dependent ROC curve analysis. Subsequently, comprehensive nomogram models based on the aforementioned clinical characteristics and MeDiff-PM were developed, and their predictive accuracy was evaluated by calibration curves and ROC curves. In the TCGA cohort, differences in MeDiff-PM gene expression and clinical characteristics, including age, sex, stage, survival time, and survival status, were also compared between the high-risk and low-risk groups.

### 4.6. Analysis of Drug Sensitivies

Drug response prediction was performed to explore potential drug response patterns associated with MeDiff-PM risk stratification. Drug sensitivity data, including the half maximal inhibitory concentration (IC50) values of 286 compounds from the Genomics of Drug Sensitivity in Cancer (GDSC) (https://www.cancerrxgene.org/ (accessed on 26 July 2023)) database, were used as the reference dataset for the oncoPredict model [62]. The TCGA CMS4 cohort and two independent CMS4 validation cohorts (GSE106584 and GSE72970) were included in this analysis. Using the R package “Oncopredict”, predicted IC50 values were estimated for patients in the three bulk cohorts [63]. Differences in predicted IC50 values between MeDiff-PM high- and low-risk groups were assessed using the Wilcoxon rank-sum test. Compounds showing consistent directional differences in predicted IC50 values between risk groups across the TCGA CMS4 cohort and independent validation cohorts were considered candidate compounds.

### 4.7. Analysis at the Methylation Level

In the TCGA cohort, the Spearman correlation between promoter methylation levels of each MeDiff-PM gene and the continuous MeDiff-PM risk scores was calculated. We then compared the differences in expression and promoter methylation levels of MeDiff-PM genes between the MeDiff-PM high- and low-risk groups of the TCGA dataset. Differential methylation analysis was performed between these two risk groups to identify differentially methylated probes (DMPs). DMPs were identified using a t-test with *p* < 0.05 and an absolute difference in mean methylation β values between groups (|Δβ|) > 0.1 as selection criteria. The hypergeometric test was used to evaluate whether identified DMPs were enriched in regulatory regions of MeDiff-PM genes.

### 4.8. Virtual Knockout Analysis of MeDiff-PM Genes

Virtual knockout of MeDiff-PM genes was performed using scTenifoldKnk to explore their potential biological relevance in CMS4 cancer cells from the CMS4 scRNA-seq data [64]. GRNs were inferred from the top 2000 highly variable genes in CMS4 cancer cells. MeDiff-PM genes not included among the selected highly variable genes were manually retained in the input matrix to ensure that all eight genes could be evaluated. Each MeDiff-PM gene was then individually knocked out in silico, followed by manifold alignment of the perturbed and original GRNs to detect genes significantly affected by each perturbation. Significantly perturbed genes were defined as those with |Z-score| > 2 and adjusted *p* value < 0.05. The overlap and recurrence of significantly perturbed genes across the eight individual knockout analyses were summarized to identify convergent transcriptional responses associated with MeDiff-PM gene perturbations.

A comprehensive summary of all software packages, versions, and key parameters used in the study is provided in Supplementary Table S4.

## Figures and Tables

**Table 1:** The results of univariate and multivariate Cox regression analyses.

		Univariate Cox			Multivariate Cox		
	Variables	HR	95% CI	*p* Value	HR	95% CI	*p* Value
TCGA							
	MeDiff-PM (High vs. low)	12.586	5.266–30.080	<0.001 ***	7.473	2.491–22.422	<0.001 ***
	Sex (Male vs. Female)	1.316	0.598–2.895	0.494	1.132	0.503–2.550	0.765
	Age	1.023	0.992–1.055	0.151	1.005	0.968–1.043	0.800
	Stage (III-IV vs. I-II)	4.450	1.671–11.852	0.003**	2.945	1.009–8.596	0.048 *
GSE72970							
	MeDiff-PM (High vs. low)	6.498	2.003–21.077	0.002 **	8.439	2.323–30.656	0.001 **
	Sex (Male vs. Female)	0.624	0.258–1.511	0.296	0.743	0.264–2.094	0.574
	Age	0.955	0.916–0.996	0.030 *	0.965	0.926–1.005	0.085
	N stage (N1-2 vs. N0)	1.800	0.240–13.492	0.567	2.402	0.281–20.538	0.423
	T stage (T3-4 vs. T1-2)	1.202	0.159–9.075	0.859	1.168	0.139–9.821	0.886

HR, hazard ratio; ‘***’, *p* < 0.001; ‘**’, *p* < 0.01; ‘*’, *p* < 0.05.

**Table 2:** The datasets used in this study.

	Series	Platform	No. Cells/Samples	CMS4
scRNA-seq	GSE200997	GPL21697	31,586/16	1491
DNA Methylation	TCGA	Illumina Infinium HumanMethylation450	394	113
Bulk	TCGA	Illumina HiSeq 2000	394	113
	GSE106584	GPL17586	156	40
	GSE72970	GPL570	124	34

## Data Availability

The data used in this study are openly available in the UCSC xena (https://xena.ucsc.edu/ (accessed on 6 June 2025)), GEO (https://www.ncbi.nlm.nih.gov/geo/ (accessed on 13 July 2023)), MsigDB (https://www.gsea-msigdb.org/gsea/msigdb (accessed on 9 January 2026)), and DisGeNET (https://www.disgenet.org/ (accessed on 8 October 2025)) databases. Images of immunohistochemistry are available from the HPA database (https://www.proteinatlas.org/ (accessed on 18 January 2026)).

## Supplementary Materials

The following supporting information can be downloaded at https://www.mdpi.com/article/10.3390/ijms27177659/s1.
